# Sensors and Fault Diagnostics in Power System

**DOI:** 10.3390/s24185999

**Published:** 2024-09-16

**Authors:** Michał Kunicki, Jan Fulneček, Pawel Rozga

**Affiliations:** 1 Department of Electrical Power Engineering and Renewable Energy, Opole University of Technology, 45-758 Opole, Poland; 2 Department of Electrical Power Engineering, VSB Technical University of Ostrava, 708 00 Ostrava, Czech Republic; jan.fulnecek@vsb.cz; 3 Institute of Electrical Power Engineering, Lodz University of Technology, Stefanowskiego 20, 90-537 Lodz, Poland; pawel.rozga@p.lodz.pl

The adequate technical condition assessment of key apparatuses is a crucial assumption in the delivery of reliable and continuous electric power to customers [1]. To meet this requirement, any fault in the power system must be detected and diagnosed as early as possible, with particular emphasis on the precision of the diagnostic process [2]. Various online and offline diagnostic methods are widely applied to the early detection of any system malfunctions [3]. Furthermore, a number of different sensors may also be applied to capture selected physical quantities that may be used to indicate the type of potential faults. A specific fault diagnostic process is typically performed by experts in the field; however, artificial intelligence (AI)-based systems are increasingly being proposed to support the decision-making process related to this task [4]. The essential step of the fault diagnostic process is signal analysis, supported by features including (but not limited to) signal processing, feature extraction, modeling, and prediction methods. Thus, the editors are pleased to present the collection of ten high-quality papers that deal with the main contemporary problems related to sensors and fault diagnostics in power system engineering. Hereunder, a brief review of each chapter of this book is presented.

In [5], a method of equivalent error is used as a criterion of the assessment of different algorithms applied for estimation of specific power system parameters. In order to indicate the best method for determining errors in measuring the synchronous parameters of the measured current or voltage waveforms, the authors propose a new form of a single error for all testing functions, which is called an equivalent error. This error is determined for each error value defined in the applicable standards for each of the selected 15 methods. According to the results, the use of the equivalent error algorithm can be very helpful in identifying a group of methods whose operation is satisfactory in terms of measurement accuracy for various types of disturbances (both in the steady state and in the dynamic state) that may occur in the power grid.

A problem of the accurate measurements and investigation of electromagnetic transients was raised in [6]. The authors proposed a capacitive electric field sensor-based measurement system to measure transient overvoltages in high-voltage substations. First, the concept and design of the measurement system is presented. Then, the design and concept are validated using tests performed in a high-voltage laboratory. Afterwards, two different calibration techniques are discussed: the simplified method (SM) and the coupling capacitance compensation (CCC) method. Finally, three recorded transients are evaluated using the calibration methods. The investigation revealed that the SM tends to overestimate the maximum overvoltage, highlighting the CCC method as a more suitable approach for calibrating transient overvoltage measurements. The proposed measurement system has been validated using various measurements and can be an efficient and flexible solution for the long-term monitoring of transient overvoltages in high-voltage substations.

In order to address the challenges of low recognition accuracy and the difficulty in effective diagnosis in traditional converter transformer voiceprint fault diagnosis, a novel method is proposed in [7]. This approach considers the impact of load factors, utilizes a multi-strategy improved Mel-Frequency Spectrum Coefficient (MFCC) for voiceprint signal feature extraction, and combines it with a temporal convolutional network for fault diagnosis. In the first step, it improves the hunter–prey optimizer (HPO) as a parameter optimization algorithm and adopts IHPO combined with variational mode decomposition (VMD) to achieve denoising of voiceprint signals. Next, the preprocessed voiceprint signal is combined with Mel filters through the Stockwell transform. To adapt to the stationary characteristics of the voiceprint signal, the processed features undergo further mid-temporal processing, ultimately resulting in the implementation of a multi-strategy improved MFCC for voiceprint signal feature extraction. Simultaneously, load signal segmentation is introduced for the diagnostic intervals, forming a joint feature vector. Finally, by using the Mish activation function to improve the temporal convolutional network, the IHPO-ITCN is proposed to adaptively optimize the size of convolutional kernels and the number of hidden layers and construct a transformer fault diagnosis model. By constructing multiple sets of comparison tests through specific examples and comparing them with the traditional voiceprint diagnostic model, our results show that the model proposed in this paper has a fault recognition accuracy as high as 99%. The recognition accuracy was significantly improved, and the training speed also shows superior performance, which can be effectively used in the field of multiple fault diagnosis of converter transformers.

In [8], the authors presented an alternative approach to the Transformer Assessment Index (TAI) by proposing a relatively simple rating method called the Exploitation Perspective Index (EPI). The method provides two numerical indicators: the first reflects the overall technical condition of the particular unit, and the second shows the condition of the unit in the context of the entire fleet. The objective of the EPI method is to support the decision-making process regarding the technical condition assessment of each of the transformers in the target population, considering not only technical but also economic aspects of transformer maintenance. Application of the method is described step by step, including input data, parametrization of the weights, and interpretation of the output results it provides. The proposed method is evaluated by two representative use cases and compared with two other methods. As a result, EPI confirms its applicability, and it has already been successfully implemented by the electric power industry. EPI can be potentially freely adopted for any transformer fleet as well as for the specific situation of the utility by adjusting the relevant parameters.

One of the well-known problems related to smart grids are outliers. They can be generated in the power system due to aging system equipment, faulty sensors, incorrect line connections, etc. The existence of these outliers will pose a threat to the safe operation of the power system, reduce the quality of the data, affect the completeness and accuracy of the data, and thus affect the monitoring analysis and control of the power system. Therefore, timely identification and treatment of outliers are essential to ensure stable and reliable operation of the power system. In [9], the minorization–maximization algorithm was used to detect and localize the outliers and an estimation of unknown parameters of the Gaussian mixture model (GMM). According to the results, the proposed algorithm provides an effective method for the handling of outliers in the power system, which helps to improve the monitoring, analyzing, and controlling ability of the power system and to ensure the stable and reliable operation of the power system.

A simplified model of the Rogowski coil is proposed in [10]. In this paper, a new user-friendly model for Rogowski coils is presented and validated. The model’s simplicity stems from utilizing information solely from the Rogowski coil datasheet. By establishing the input/output relationship, the output of the Rogowski coil is obtained. The effectiveness and accuracy of the proposed model are tested using both simulations and commercially available Rogowski coils. The results confirm that the model is simple, accurate, and easily implementable in various simulation environments for a wide range of applications and purposes.

System stability deterioration in microgrids commonly occurs due to unpredictable faults and equipment malfunctions. Recently, robust control techniques have been used in microgrid systems to address these difficulties. One of these alternative control strategies is proposed in [11]. The suggested approach can be used to maintain system stability in the presence of flaws, such as faulty actuators and sensors, as well as component failures. The proposed control is effective when the fault is never recognized (or when the fault is not being precisely known, and some ambiguity in the fault may be interpreted as uncertainty in the system’s dynamics following the fault). The design is built around a derived sufficient condition in the context of linear matrix inequalities (LMIs) and the attractive ellipsoid technique. The ellipsoidal stabilization idea is to bring the state trajectories into a small region including the origin (an ellipsoid with minimum volume), and the trajectories will not leave the ellipsoid in the future. Finally, computational studies on a DC microgrid system are carried out to assess the effectiveness of the proposed fault-tolerant control approach. When compared with previous studies, the simulation results demonstrate that the proposed control technique can significantly enhance the reliability and efficiency of DC microgrid systems.

In [12], a new method for condition assessment of natural ester–mineral oil mixtures due to transformer retrofilling via sensing dielectric properties is announced. In this study, two accelerated aging processes were applied to mineral oil for 6 and 12 days to simulate mineral oil in service for 6 and 12 years. Moreover, these aged oils were mixed with 80% and 90% fresh natural ester oil. The dielectric strength, relative permittivity, and dissipation factor were sensed using an LCR meter and oil tester devices for all prepared samples to support the condition assessment performance of the oil mixtures. In addition, the electric field distribution was analyzed for a power transformer using the oil mixtures. Furthermore, the dynamic viscosity was measured for all insulating oil samples at different temperatures. From the obtained results, the sample obtained by mixing 90% natural ester oil with 10% mineral oil aged for 6 days is considered superior and achieves an improvement in dielectric strength and relative permittivity by approximately 43% and 48%, respectively, compared to fresh mineral oil. However, the dissipation factor was increased by approximately 20% but was at an acceptable limit. On the other hand, for the same oil sample, due to the higher molecular weight of the natural ester oil, the viscosities of all mixtures were at a higher level than the mineral oil.

A proposal of a novel test tool for diagnosis of contact resistance and measurement of selected types of conductive materials is presented in [13]. It is obvious that contact resistance is a fundamental criterion in the design of an electrical contact or contact system. The value of the contact resistance depends on the material used, the value of the applied force, the type of contact, and, last but not least, the quality of the surface and chemical layers. In this paper, an initial diagnosis of the contact material is performed based on the determination of the sample’s specific resistivity by the four-wire method and the evaluation of the measurement uncertainty. The work is followed by the design of a testing device that uses crossed bars to measure the change in contact resistance as a function of the magnitude of the applied force. An analysis of the sample mounting method is performed here using FEM simulations of the current field and shows the interaction between the holder and the sample in terms of current line transfer. The proposed system is then used for experimental measurements of the material-dependent coefficient K_C_ for verification of existing or newly developed materials in electrical engineering, where the values of the K_C_ coefficient are not known.

The location of the grounding grid conductors is critical for performing corrosion diagnosis and maintenance work. An improved magnetic field differential method to locate the unknown grounding grid based on truncation errors and the round-off error analysis is presented in [14]. It was proven that a different order of the magnetic field derivative can be used to determine the position of the grounding conductor according to the peak value of the derivative. Due to the accumulative error of higher differentiation, the truncation error and rounding error were used to analyze the cumulative error and to determine the optimal step size to measure and calculate the higher differentiation. The possible range and probability distribution of the two kinds of errors at each order are described, and the index of peak position error was derived, which can be used to locate the grounding conductor in the power substation.
